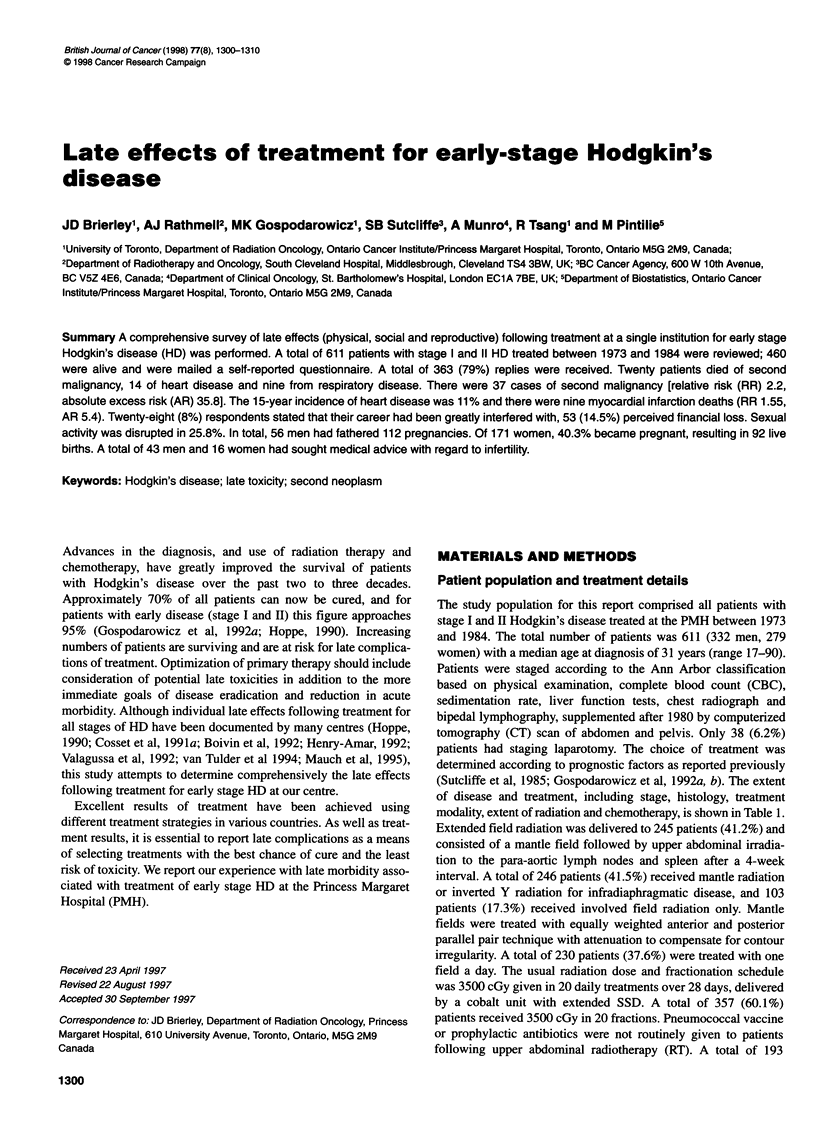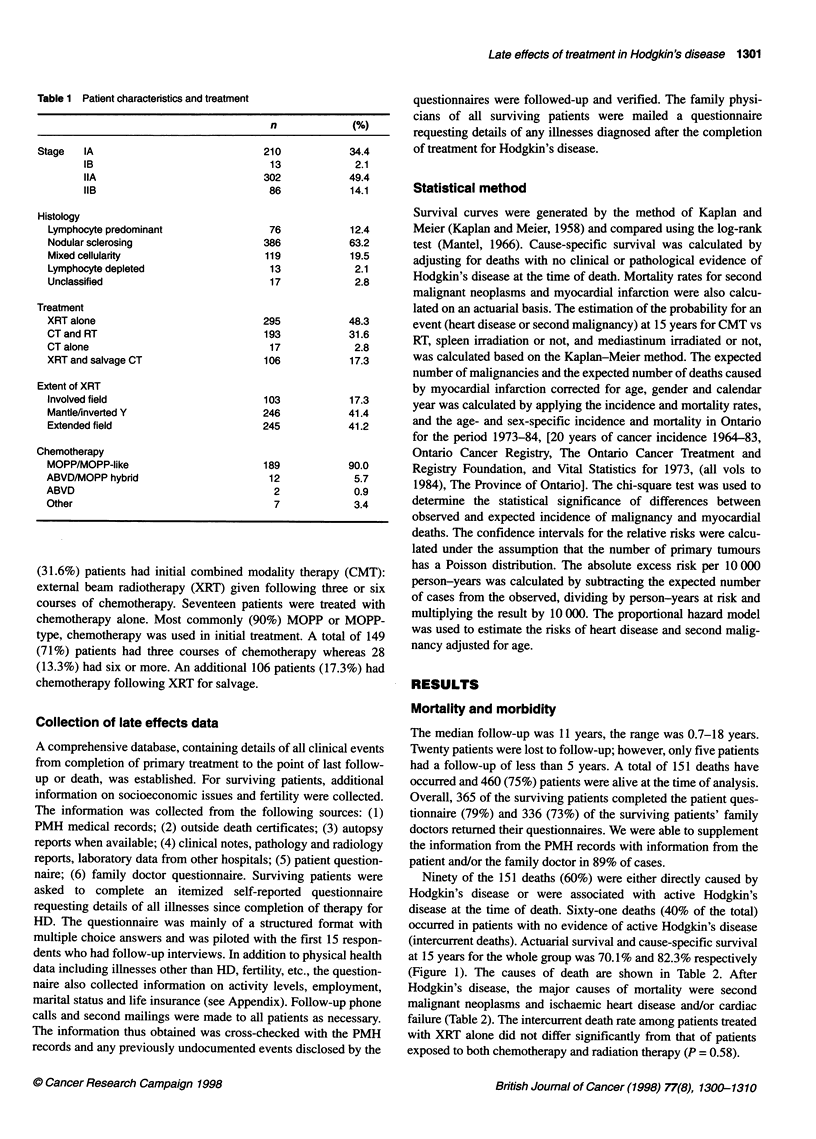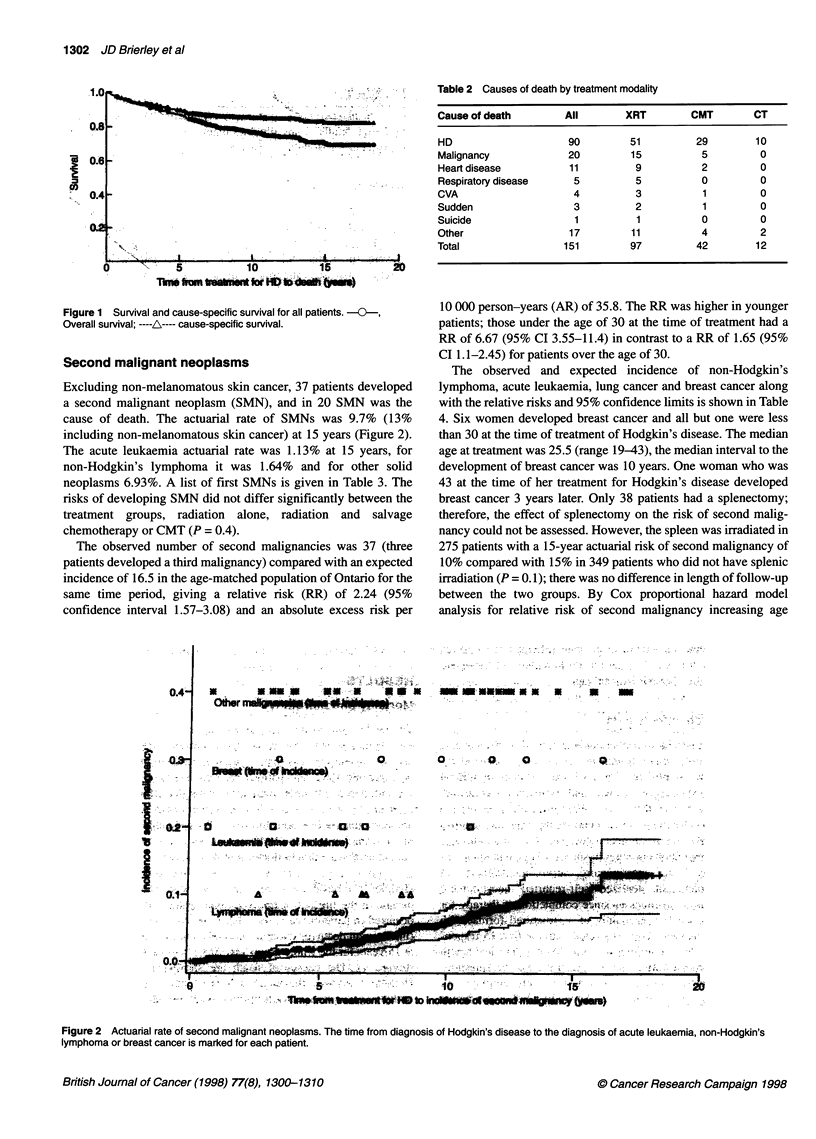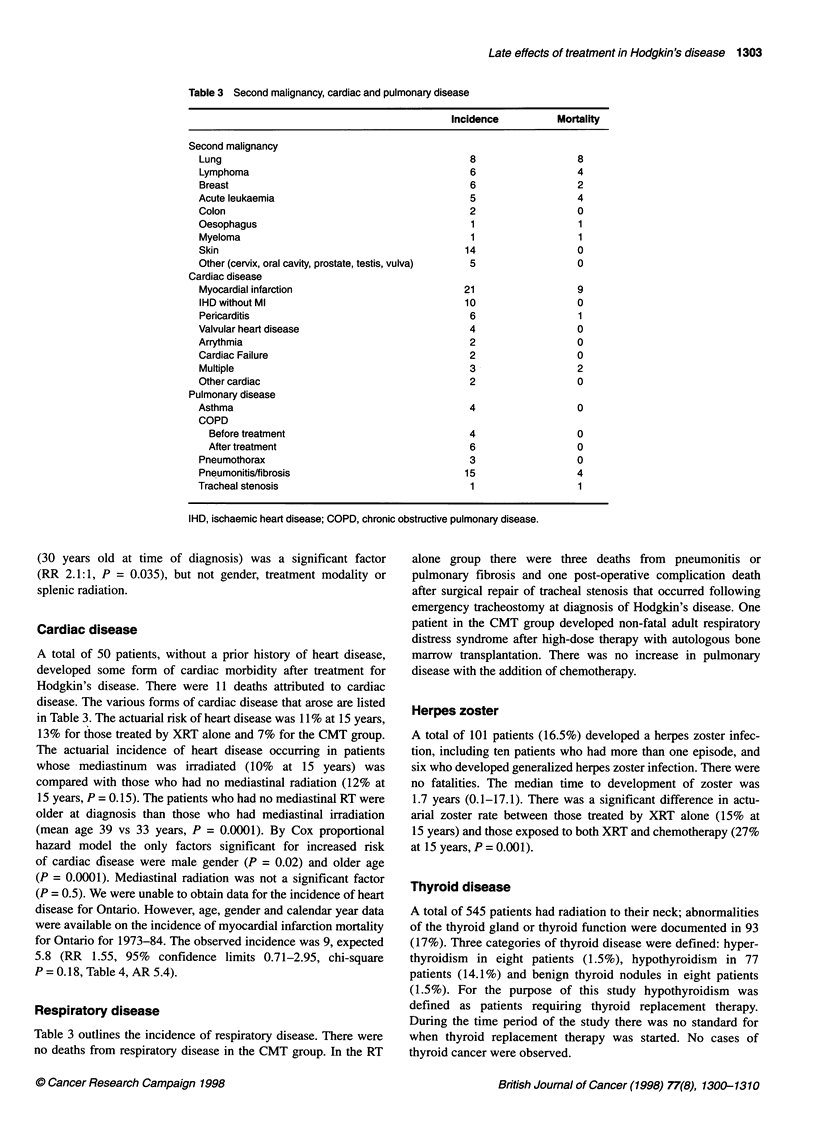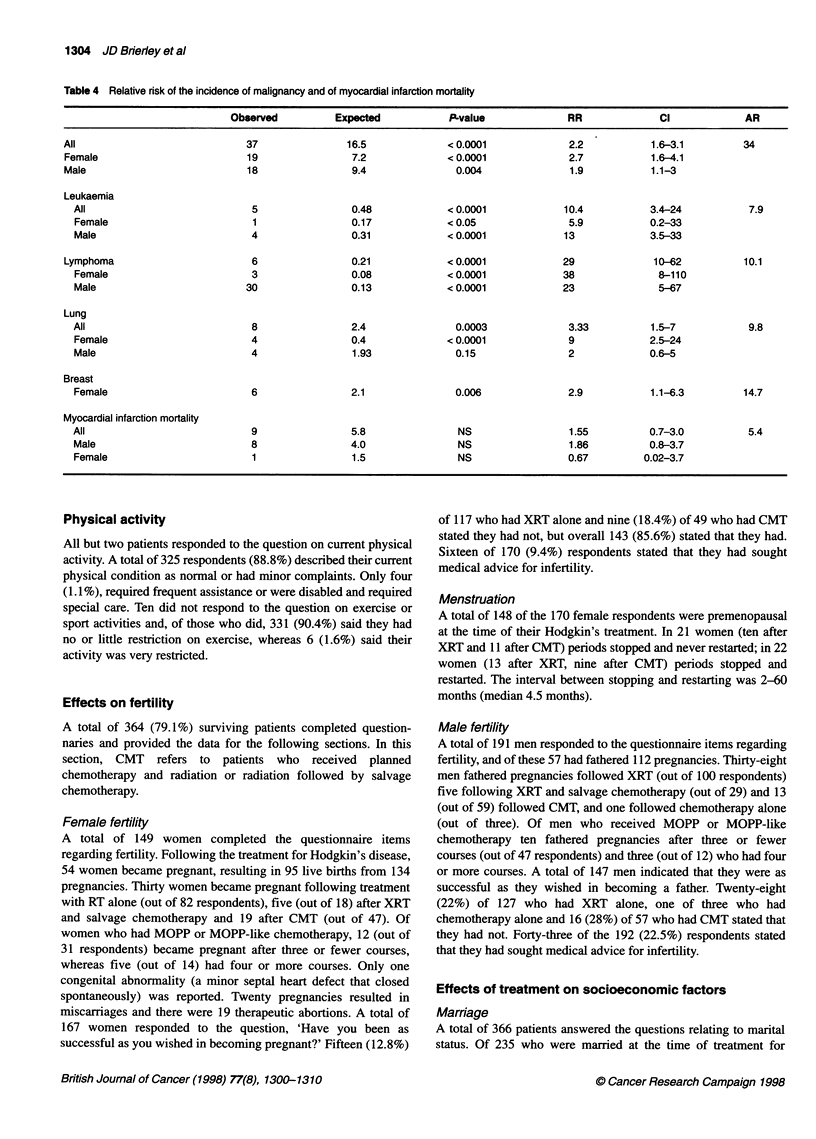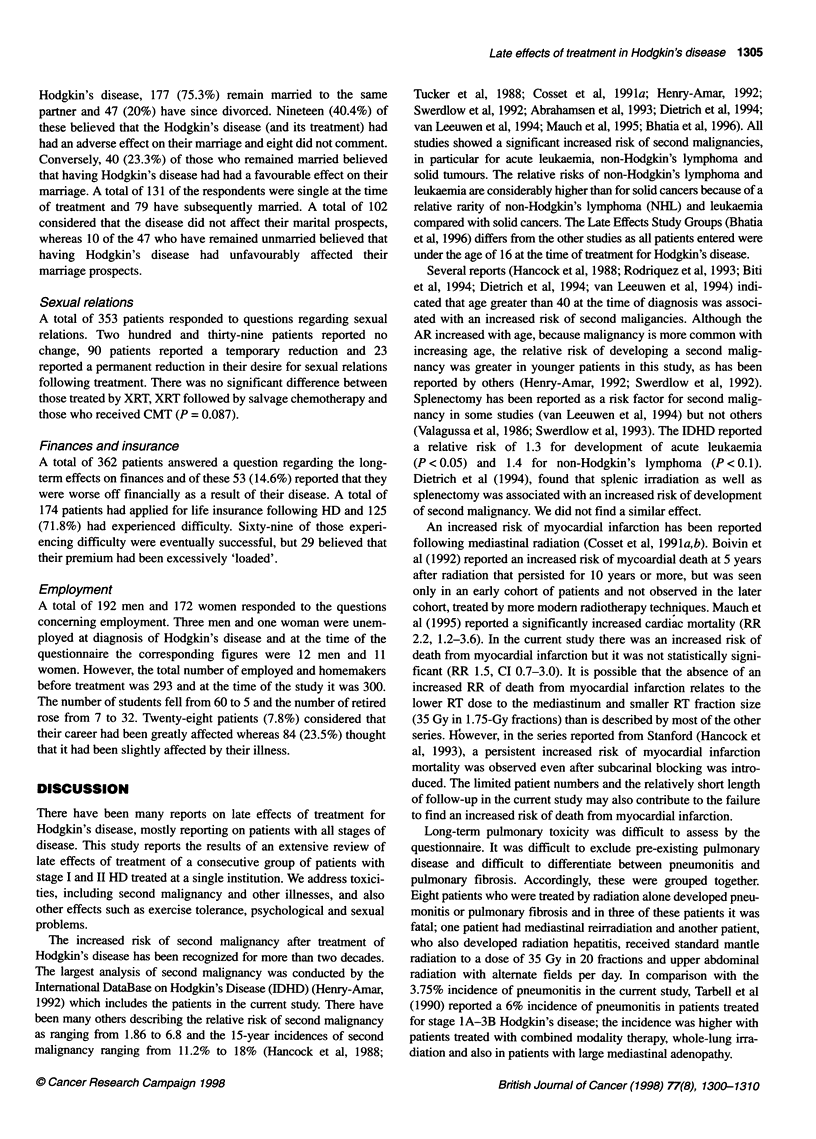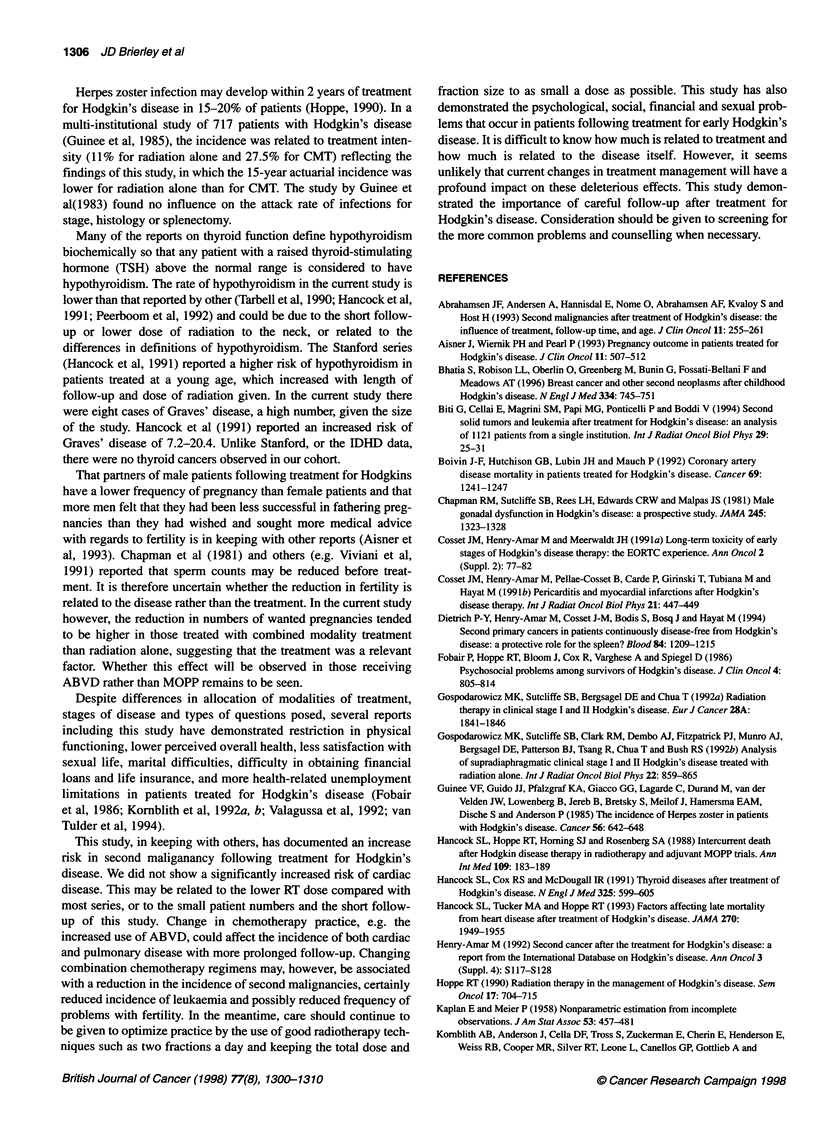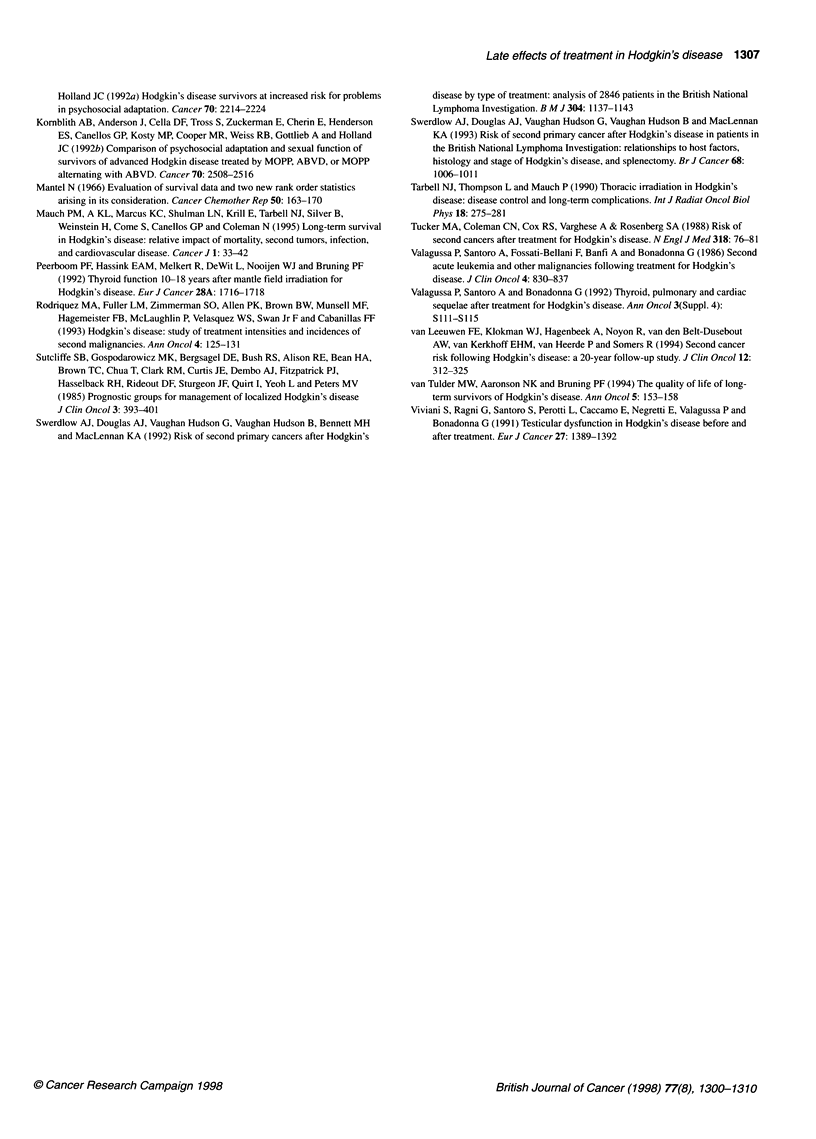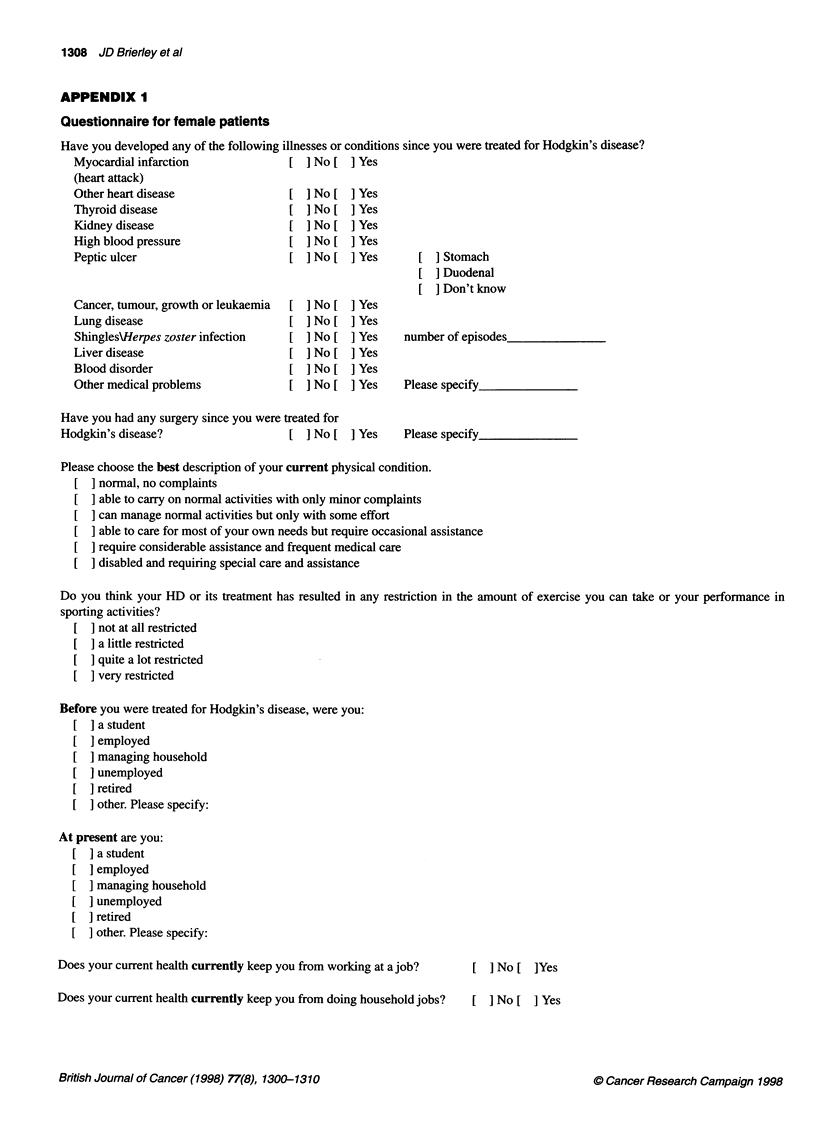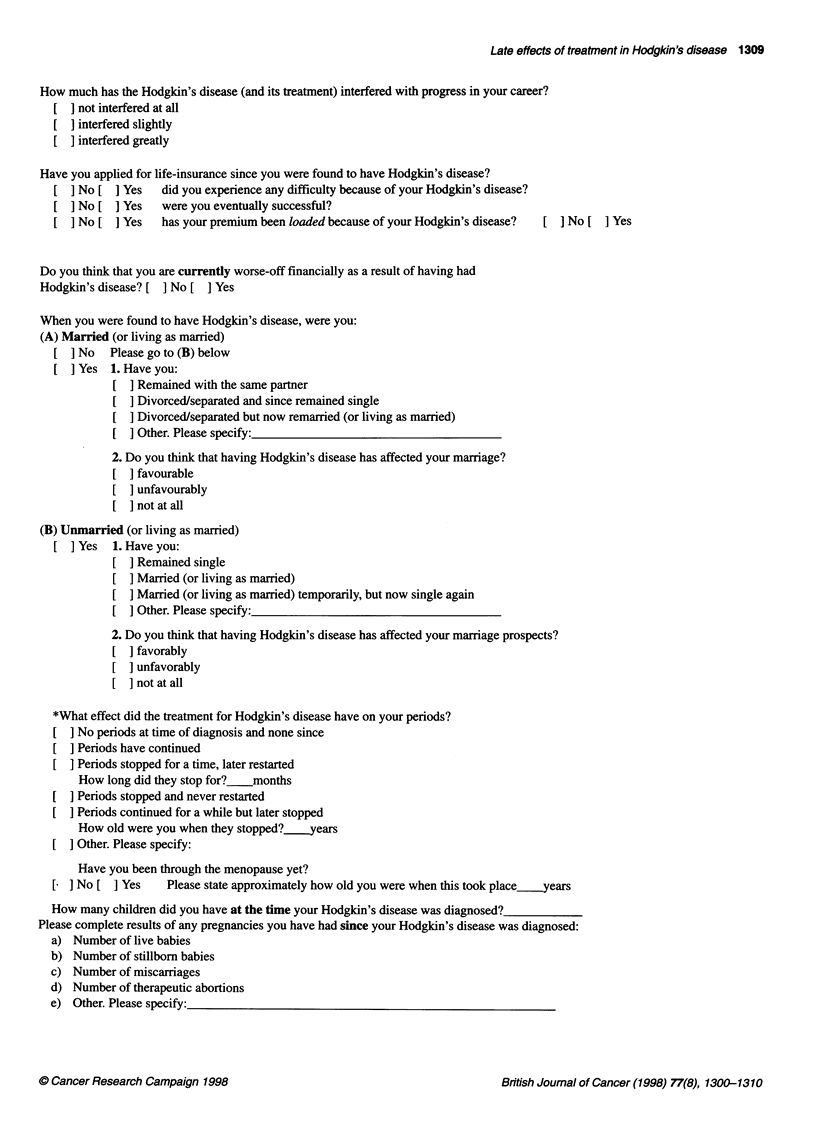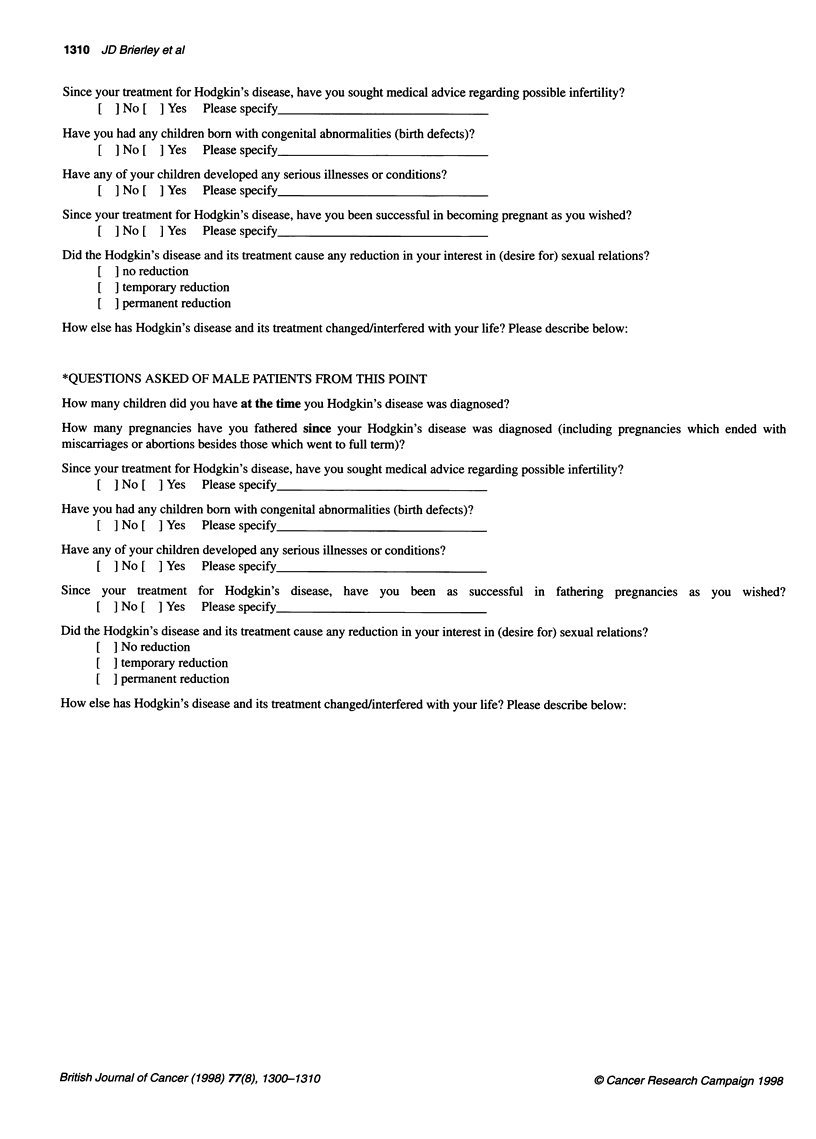# Late effects of treatment for early-stage Hodgkin's disease.

**DOI:** 10.1038/bjc.1998.217

**Published:** 1998-04

**Authors:** J. D. Brierley, A. J. Rathmell, M. K. Gospodarowicz, S. B. Sutcliffe, A. Munro, R. Tsang, M. Pintilie

**Affiliations:** University of Toronto, Department of Radiation Oncology, Ontario Cancer Institute/Princess Margaret Hospital, Canada.

## Abstract

A comprehensive survey of late effects (physical, social and reproductive) following treatment at a single institution for early stage Hodgkin's disease (HD) was performed. A total of 611 patients with stage I and II HD treated between 1973 and 1984 were reviewed; 460 were alive and were mailed a self-reported questionnaire. A total of 363 (79%) replies were received. Twenty patients died of second malignancy, 14 of heart disease and nine from respiratory disease. There were 37 cases of second malignancy [relative risk (RR) 2.2, absolute excess risk (AR) 35.8]. The 15-year incidence of heart disease was 11% and there were nine myocardial infarction deaths (RR 1.55, AR 5.4). Twenty-eight (8%) respondents stated that their career had been greatly interfered with, 53 (14.5%) perceived financial loss. Sexual activity was disrupted in 25.8%. In total, 56 men had fathered 112 pregnancies. Of 171 women, 40.3% became pregnant, resulting in 92 live births. A total of 43 men and 16 women had sought medical advice with regard to infertility.


British Journal of Cancer (1998) 77(8), 1300–1310
© 1998 Cancer Research Campaign

### Late effects of treatment for early-stage Hodgkin's disease

**JD Brierley[1], AJ Rathmell[2], MK Gospodarowicz[1], SB Sutcliffe[3], A Munro[4], R Tsang[1] and M Pintilie[5]**

[1]University of Toronto, Department of Radiation Oncology, Ontario Cancer Institute/Princess Margaret Hospital, Toronto, Ontario M5G 2M9, Canada; [2]Department of Radiotherapy and Oncology, South Cleveland Hospital, Middlesbrough, Cleveland TS4 3BW, UK; [3]BC Cancer Agency, 600 W 10th Avenue, BC V5Z 4E6, Canada; [4]Department of Clinical Oncology, St. Bartholomew's Hospital, London EC1A 7BE, UK; [5]Department of Biostatistics, Ontario Cancer Institute/Princess Margaret Hospital, Toronto, Ontario M5G 2M9, Canada

**Summary** A comprehensive survey of late effects (physical, social and reproductive) following treatment at a single institution for early stage Hodgkin's disease (HD) was performed. A total of 611 patients with stage I and II HD treated between 1973 and 1984 were reviewed; 460 were alive and were mailed a self-reported questionnaire. A total of 363 (79%) replies were received. Twenty patients died of second malignancy, 14 of heart disease and nine from respiratory disease. There were 37 cases of second malignancy [relative risk (RR) 2.2, absolute excess risk (AR) 35.8]. The 15-year incidence of heart disease was 11% and there were nine myocardial infarction deaths (RR 1.55, AR 5.4). Twenty-eight (8%) respondents stated that their career had been greatly interfered with, 53 (14.5%) perceived financial loss. Sexual activity was disrupted in 25.8%. In total, 56 men had fathered 112 pregnancies. Of 171 women, 40.3% became pregnant, resulting in 92 live births. A total of 43 men and 16 women had sought medical advice with regard to infertility.

**Keywords:** Hodgkin's disease; late toxicity; second neoplasm

Advances in the diagnosis, and use of radiation therapy and chemotherapy, have greatly improved the survival of patients with Hodgkin's disease over the past two to three decades. Approximately 70% of all patients can now be cured, and for patients with early disease (stage I and II) this figure approaches 95% (Gospodarowicz et al, 1992*a*; Hoppe, 1990). Increasing numbers of patients are surviving and are at risk for late complications of treatment. Optimization of primary therapy should include consideration of potential late toxicities in addition to the more immediate goals of disease eradication and reduction in acute morbidity. Although individual late effects following treatment for all stages of HD have been documented by many centres (Hoppe, 1990; Cosset et al, 1991*a*; Boivin et al, 1992; Henry-Amar, 1992; Valagussa et al, 1992; van Tulder et al 1994; Mauch et al, 1995), this study attempts to determine comprehensively the late effects following treatment for early stage HD at our centre.

Excellent results of treatment have been achieved using different treatment strategies in various countries. As well as treatment results, it is essential to report late complications as a means of selecting treatments with the best chance of cure and the least risk of toxicity. We report our experience with late morbidity associated with treatment of early stage HD at the Princess Margaret Hospital (PMH).

*Received 23 April 1997*
*Revised 22 August 1997*
*Accepted 30 September 1997*

*Correspondence to:* JD Brierley, Department of Radiation Oncology, Princess Margaret Hospital, 610 University Avenue, Toronto, Ontario, M5G 2M9 Canada

### MATERIALS AND METHODS

### Patient population and treatment details

The study population for this report comprised all patients with stage I and II Hodgkin's disease treated at the PMH between 1973 and 1984. The total number of patients was 611 (332 men, 279 women) with a median age at diagnosis of 31 years (range 17–90). Patients were staged according to the Ann Arbor classification based on physical examination, complete blood count (CBC), sedimentation rate, liver function tests, chest radiograph and bipedal lymphography, supplemented after 1980 by computerized tomography (CT) scan of abdomen and pelvis. Only 38 (6.2%) patients had staging laparotomy. The choice of treatment was determined according to prognostic factors as reported previously (Sutcliffe et al, 1985; Gospodarowicz et al, 1992*a, b*). The extent of disease and treatment, including stage, histology, treatment modality, extent of radiation and chemotherapy, is shown in Table 1. Extended field radiation was delivered to 245 patients (41.2%) and consisted of a mantle field followed by upper abdominal irradiation to the para-aortic lymph nodes and spleen after a 4-week interval. A total of 246 patients (41.5%) received mantle radiation or inverted Y radiation for infradiaphragmatic disease, and 103 patients (17.3%) received involved field radiation only. Mantle fields were treated with equally weighted anterior and posterior parallel pair technique with attenuation to compensate for contour irregularity. A total of 230 patients (37.6%) were treated with one field a day. The usual radiation dose and fractionation schedule was 3500 cGy given in 20 daily treatments over 28 days, delivered by a cobalt unit with extended SSD. A total of 357 (60.1%) patients received 3500 cGy in 20 fractions. Pneumococcal vaccine or prophylactic antibiotics were not routinely given to patients following upper abdominal radiotherapy (RT). A total of 193

**Table 1** Patient characteristics and treatment

| | | n | (%) |
|---|---|---|---|
| Stage | IA | 210 | 34.4 |
| | IB | 13 | 2.1 |
| | IIA | 302 | 49.4 |
| | IIB | 86 | 14.1 |
| Histology | | | |
| Lymphocyte predominant | | 76 | 12.4 |
| Nodular sclerosing | | 386 | 63.2 |
| Mixed cellularity | | 119 | 19.5 |
| Lymphocyte depleted | | 13 | 2.1 |
| Unclassified | | 17 | 2.8 |
| Treatment | | | |
| XRT alone | | 295 | 48.3 |
| CT and RT | | 193 | 31.6 |
| CT alone | | 17 | 2.8 |
| XRT and salvage CT | | 106 | 17.3 |
| Extent of XRT | | | |
| Involved field | | 103 | 17.3 |
| Mantle/inverted Y | | 246 | 41.4 |
| Extended field | | 245 | 41.2 |
| Chemotherapy | | | |
| MOPP/MOPP-like | | 189 | 90.0 |
| ABVD/MOPP hybrid | | 12 | 5.7 |
| ABVD | | 2 | 0.9 |
| Other | | 7 | 3.4 |

(31.6%) patients had initial combined modality therapy (CMT): external beam radiotherapy (XRT) given following three or six courses of chemotherapy. Seventeen patients were treated with chemotherapy alone. Most commonly (90%) MOPP or MOPP-type, chemotherapy was used in initial treatment. A total of 149 (71%) patients had three courses of chemotherapy whereas 28 (13.3%) had six or more. An additional 106 patients (17.3%) had chemotherapy following XRT for salvage.

### Collection of late effects data

A comprehensive database, containing details of all clinical events from completion of primary treatment to the point of last follow-up or death, was established. For surviving patients, additional information on socioeconomic issues and fertility were collected. The information was collected from the following sources: (1) PMH medical records; (2) outside death certificates; (3) autopsy reports when available; (4) clinical notes, pathology and radiology reports, laboratory data from other hospitals; (5) patient questionnaire; (6) family doctor questionnaire. Surviving patients were asked to complete an itemized self-reported questionnaire requesting details of all illnesses since completion of therapy for HD. The questionnaire was mainly of a structured format with multiple choice answers and was piloted with the first 15 respondents who had follow-up interviews. In addition to physical health data including illnesses other than HD, fertility, etc., the questionnaire also collected information on activity levels, employment, marital status and life insurance (see Appendix). Follow-up phone calls and second mailings were made to all patients as necessary. The information thus obtained was cross-checked with the PMH records and any previously undocumented events disclosed by the questionnaires were followed-up and verified. The family physicians of all surviving patients were mailed a questionnaire requesting details of any illnesses diagnosed after the completion of treatment for Hodgkin's disease.

### Statistical method

Survival curves were generated by the method of Kaplan and Meier (Kaplan and Meier, 1958) and compared using the log-rank test (Mantel, 1966). Cause-specific survival was calculated by adjusting for deaths with no clinical or pathological evidence of Hodgkin's disease at the time of death. Mortality rates for second malignant neoplasms and myocardial infarction were also calculated on an actuarial basis. The estimation of the probability for an event (heart disease or second malignancy) at 15 years for CMT vs RT, spleen irradiation or not, and mediastinum irradiated or not, was calculated based on the Kaplan–Meier method. The expected number of malignancies and the expected number of deaths caused by myocardial infarction corrected for age, gender and calendar year was calculated by applying the incidence and mortality rates, and the age- and sex-specific incidence and mortality in Ontario for the period 1973–84, [20 years of cancer incidence 1964–83, Ontario Cancer Registry, The Ontario Cancer Treatment and Registry Foundation, and Vital Statistics for 1973, (all vols to 1984), The Province of Ontario]. The chi-square test was used to determine the statistical significance of differences between observed and expected incidence of malignancy and myocardial deaths. The confidence intervals for the relative risks were calculated under the assumption that the number of primary tumours has a Poisson distribution. The absolute excess risk per 10 000 person–years was calculated by subtracting the expected number of cases from the observed, dividing by person–years at risk and multiplying the result by 10 000. The proportional hazard model was used to estimate the risks of heart disease and second malignancy adjusted for age.

### RESULTS

### Mortality and morbidity

The median follow-up was 11 years, the range was 0.7–18 years. Twenty patients were lost to follow-up; however, only five patients had a follow-up of less than 5 years. A total of 151 deaths have occurred and 460 (75%) patients were alive at the time of analysis. Overall, 365 of the surviving patients completed the patient questionnaire (79%) and 336 (73%) of the surviving patients' family doctors returned their questionnaires. We were able to supplement the information from the PMH records with information from the patient and/or the family doctor in 89% of cases.

Ninety of the 151 deaths (60%) were either directly caused by Hodgkin's disease or were associated with active Hodgkin's disease at the time of death. Sixty-one deaths (40% of the total) occurred in patients with no evidence of active Hodgkin's disease (intercurrent deaths). Actuarial survival and cause-specific survival at 15 years for the whole group was 70.1% and 82.3% respectively (Figure 1). The causes of death are shown in Table 2. After Hodgkin's disease, the major causes of mortality were second malignant neoplasms and ischaemic heart disease and/or cardiac failure (Table 2). The intercurrent death rate among patients treated with XRT alone did not differ significantly from that of patients exposed to both chemotherapy and radiation therapy ($P = 0.58$).

© Cancer Research Campaign 1998

**Figure 1** Survival and cause-specific survival for all patients. —○—, Overall survival; ----△---- cause-specific survival.

**Table 2** Causes of death by treatment modality

| Cause of death | All | XRT | CMT | CT |
|---|---|---|---|---|
| HD | 90 | 51 | 29 | 10 |
| Malignancy | 20 | 15 | 5 | 0 |
| Heart disease | 11 | 9 | 2 | 0 |
| Respiratory disease | 5 | 5 | 0 | 0 |
| CVA | 4 | 3 | 1 | 0 |
| Sudden | 3 | 2 | 1 | 0 |
| Suicide | 1 | 1 | 0 | 0 |
| Other | 17 | 11 | 4 | 2 |
| Total | 151 | 97 | 42 | 12 |

### Second malignant neoplasms

Excluding non-melanomatous skin cancer, 37 patients developed a second malignant neoplasm (SMN), and in 20 SMN was the cause of death. The actuarial rate of SMNs was 9.7% (13% including non-melanomatous skin cancer) at 15 years (Figure 2). The acute leukaemia actuarial rate was 1.13% at 15 years, for non-Hodgkin's lymphoma it was 1.64% and for other solid neoplasms 6.93%. A list of first SMNs is given in Table 3. The risks of developing SMN did not differ significantly between the treatment groups, radiation alone, radiation and salvage chemotherapy or CMT ($P = 0.4$).

The observed number of second malignancies was 37 (three patients developed a third malignancy) compared with an expected incidence of 16.5 in the age-matched population of Ontario for the same time period, giving a relative risk (RR) of 2.24 (95% confidence interval 1.57–3.08) and an absolute excess risk per 10 000 person–years (AR) of 35.8. The RR was higher in younger patients; those under the age of 30 at the time of treatment had a RR of 6.67 (95% CI 3.55–11.4) in contrast to a RR of 1.65 (95% CI 1.1–2.45) for patients over the age of 30.

The observed and expected incidence of non-Hodgkin's lymphoma, acute leukaemia, lung cancer and breast cancer along with the relative risks and 95% confidence limits is shown in Table 4. Six women developed breast cancer and all but one were less than 30 at the time of treatment of Hodgkin's disease. The median age at treatment was 25.5 (range 19–43), the median interval to the development of breast cancer was 10 years. One woman who was 43 at the time of her treatment for Hodgkin's disease developed breast cancer 3 years later. Only 38 patients had a splenectomy; therefore, the effect of splenectomy on the risk of second malignancy could not be assessed. However, the spleen was irradiated in 275 patients with a 15-year actuarial risk of second malignancy of 10% compared with 15% in 349 patients who did not have splenic irradiation ($P = 0.1$); there was no difference in length of follow-up between the two groups. By Cox proportional hazard model analysis for relative risk of second malignancy increasing age

**Figure 2** Actuarial rate of second malignant neoplasms. The time from diagnosis of Hodgkin's disease to the diagnosis of acute leukaemia, non-Hodgkin's lymphoma or breast cancer is marked for each patient.

© Cancer Research Campaign 1998

**Table 3** Second malignancy, cardiac and pulmonary disease

| | Incidence | Mortality |
|---|---|---|
| Second malignancy | | |
| Lung | 8 | 8 |
| Lymphoma | 6 | 4 |
| Breast | 6 | 2 |
| Acute leukaemia | 5 | 4 |
| Colon | 2 | 0 |
| Oesophagus | 1 | 1 |
| Myeloma | 1 | 1 |
| Skin | 14 | 0 |
| Other (cervix, oral cavity, prostate, testis, vulva) | 5 | 0 |
| Cardiac disease | | |
| Myocardial infarction | 21 | 9 |
| IHD without MI | 10 | 0 |
| Pericarditis | 6 | 1 |
| Valvular heart disease | 4 | 0 |
| Arrythmia | 2 | 0 |
| Cardiac Failure | 2 | 0 |
| Multiple | 3 | 2 |
| Other cardiac | 2 | 0 |
| Pulmonary disease | | |
| Asthma | 4 | 0 |
| COPD | | |
| Before treatment | 4 | 0 |
| After treatment | 6 | 0 |
| Pneumothorax | 3 | 0 |
| Pneumonitis/fibrosis | 15 | 4 |
| Tracheal stenosis | 1 | 1 |

IHD, ischaemic heart disease; COPD, chronic obstructive pulmonary disease.

(30 years old at time of diagnosis) was a significant factor (RR 2.1:1, $P$ = 0.035), but not gender, treatment modality or splenic radiation.

### Cardiac disease

A total of 50 patients, without a prior history of heart disease, developed some form of cardiac morbidity after treatment for Hodgkin's disease. There were 11 deaths attributed to cardiac disease. The various forms of cardiac disease that arose are listed in Table 3. The actuarial risk of heart disease was 11% at 15 years, 13% for those treated by XRT alone and 7% for the CMT group. The actuarial incidence of heart disease occurring in patients whose mediastinum was irradiated (10% at 15 years) was compared with those who had no mediastinal radiation (12% at 15 years, $P$ = 0.15). The patients who had no mediastinal RT were older at diagnosis than those who had mediastinal irradiation (mean age 39 vs 33 years, $P$ = 0.0001). By Cox proportional hazard model the only factors significant for increased risk of cardiac disease were male gender ($P$ = 0.02) and older age ($P$ = 0.0001). Mediastinal radiation was not a significant factor ($P$ = 0.5). We were unable to obtain data for the incidence of heart disease for Ontario. However, age, gender and calendar year data were available on the incidence of myocardial infarction mortality for Ontario for 1973–84. The observed incidence was 9, expected 5.8 (RR 1.55, 95% confidence limits 0.71–2.95, chi-square $P$ = 0.18, Table 4, AR 5.4).

### Respiratory disease

Table 3 outlines the incidence of respiratory disease. There were no deaths from respiratory disease in the CMT group. In the RT alone group there were three deaths from pneumonitis or pulmonary fibrosis and one post-operative complication death after surgical repair of tracheal stenosis that occurred following emergency tracheostomy at diagnosis of Hodgkin's disease. One patient in the CMT group developed non-fatal adult respiratory distress syndrome after high-dose therapy with autologous bone marrow transplantation. There was no increase in pulmonary disease with the addition of chemotherapy.

### Herpes zoster

A total of 101 patients (16.5%) developed a herpes zoster infection, including ten patients who had more than one episode, and six who developed generalized herpes zoster infection. There were no fatalities. The median time to development of zoster was 1.7 years (0.1–17.1). There was a significant difference in actuarial zoster rate between those treated by XRT alone (15% at 15 years) and those exposed to both XRT and chemotherapy (27% at 15 years, $P$ = 0.001).

### Thyroid disease

A total of 545 patients had radiation to their neck; abnormalities of the thyroid gland or thyroid function were documented in 93 (17%). Three categories of thyroid disease were defined: hyperthyroidism in eight patients (1.5%), hypothyroidism in 77 patients (14.1%) and benign thyroid nodules in eight patients (1.5%). For the purpose of this study hypothyroidism was defined as patients requiring thyroid replacement therapy. During the time period of the study there was no standard for when thyroid replacement therapy was started. No cases of thyroid cancer were observed.

© Cancer Research Campaign 1998

**Table 4** Relative risk of the incidence of malignancy and of myocardial infarction mortality

| | Observed | Expected | P-value | RR | CI | AR |
|---|---|---|---|---|---|---|
| All | 37 | 16.5 | < 0.0001 | 2.2 | 1.6–3.1 | 34 |
| Female | 19 | 7.2 | < 0.0001 | 2.7 | 1.6–4.1 | |
| Male | 18 | 9.4 | 0.004 | 1.9 | 1.1–3 | |
| Leukaemia | | | | | | |
| All | 5 | 0.48 | < 0.0001 | 10.4 | 3.4–24 | 7.9 |
| Female | 1 | 0.17 | < 0.05 | 5.9 | 0.2–33 | |
| Male | 4 | 0.31 | < 0.0001 | 13 | 3.5–33 | |
| Lymphoma | 6 | 0.21 | < 0.0001 | 29 | 10–62 | 10.1 |
| Female | 3 | 0.08 | < 0.0001 | 38 | 8–110 | |
| Male | 30 | 0.13 | < 0.0001 | 23 | 5–67 | |
| Lung | | | | | | |
| All | 8 | 2.4 | 0.0003 | 3.33 | 1.5–7 | 9.8 |
| Female | 4 | 0.4 | < 0.0001 | 9 | 2.5–24 | |
| Male | 4 | 1.93 | 0.15 | 2 | 0.6–5 | |
| Breast | | | | | | |
| Female | 6 | 2.1 | 0.006 | 2.9 | 1.1–6.3 | 14.7 |
| Myocardial infarction mortality | | | | | | |
| All | 9 | 5.8 | NS | 1.55 | 0.7–3.0 | 5.4 |
| Male | 8 | 4.0 | NS | 1.86 | 0.8–3.7 | |
| Female | 1 | 1.5 | NS | 0.67 | 0.02–3.7 | |

### Physical activity

All but two patients responded to the question on current physical activity. A total of 325 respondents (88.8%) described their current physical condition as normal or had minor complaints. Only four (1.1%), required frequent assistance or were disabled and required special care. Ten did not respond to the question on exercise or sport activities and, of those who did, 331 (90.4%) said they had no or little restriction on exercise, whereas 6 (1.6%) said their activity was very restricted.

### Effects on fertility

A total of 364 (79.1%) surviving patients completed questionnaries and provided the data for the following sections. In this section, CMT refers to patients who received planned chemotherapy and radiation or radiation followed by salvage chemotherapy.

### *Female fertility*

A total of 149 women completed the questionnaire items regarding fertility. Following the treatment for Hodgkin's disease, 54 women became pregnant, resulting in 95 live births from 134 pregnancies. Thirty women became pregnant following treatment with RT alone (out of 82 respondents), five (out of 18) after XRT and salvage chemotherapy and 19 after CMT (out of 47). Of women who had MOPP or MOPP-like chemotherapy, 12 (out of 31 respondents) became pregnant after three or fewer courses, whereas five (out of 14) had four or more courses. Only one congenital abnormality (a minor septal heart defect that closed spontaneously) was reported. Twenty pregnancies resulted in miscarriages and there were 19 therapeutic abortions. A total of 167 women responded to the question, 'Have you been as successful as you wished in becoming pregnant?' Fifteen (12.8%) of 117 who had XRT alone and nine (18.4%) of 49 who had CMT stated they had not, but overall 143 (85.6%) stated that they had. Sixteen of 170 (9.4%) respondents stated that they had sought medical advice for infertility.

### *Menstruation*

A total of 148 of the 170 female respondents were premenopausal at the time of their Hodgkin's treatment. In 21 women (ten after XRT and 11 after CMT) periods stopped and never restarted; in 22 women (13 after XRT, nine after CMT) periods stopped and restarted. The interval between stopping and restarting was 2–60 months (median 4.5 months).

### *Male fertility*

A total of 191 men responded to the questionnaire items regarding fertility, and of these 57 had fathered 112 pregnancies. Thirty-eight men fathered pregnancies followed XRT (out of 100 respondents) five following XRT and salvage chemotherapy (out of 29) and 13 (out of 59) followed CMT, and one followed chemotherapy alone (out of three). Of men who received MOPP or MOPP-like chemotherapy ten fathered pregnancies after three or fewer courses (out of 47 respondents) and three (out of 12) who had four or more courses. A total of 147 men indicated that they were as successful as they wished in becoming a father. Twenty-eight (22%) of 127 who had XRT alone, one of three who had chemotherapy alone and 16 (28%) of 57 who had CMT stated that they had not. Forty-three of the 192 (22.5%) respondents stated that they had sought medical advice for infertility.

### Effects of treatment on socioeconomic factors

### *Marriage*

A total of 366 patients answered the questions relating to marital status. Of 235 who were married at the time of treatment for

 © Cancer Research Campaign 1998

Hodgkin's disease, 177 (75.3%) remain married to the same partner and 47 (20%) have since divorced. Nineteen (40.4%) of these believed that the Hodgkin's disease (and its treatment) had had an adverse effect on their marriage and eight did not comment. Conversely, 40 (23.3%) of those who remained married believed that having Hodgkin's disease had had a favourable effect on their marriage. A total of 131 of the respondents were single at the time of treatment and 79 have subsequently married. A total of 102 considered that the disease did not affect their marital prospects, whereas 10 of the 47 who have remained unmarried believed that having Hodgkin's disease had unfavourably affected their marriage prospects.

### *Sexual relations*

A total of 353 patients responded to questions regarding sexual relations. Two hundred and thirty-nine patients reported no change, 90 patients reported a temporary reduction and 23 reported a permanent reduction in their desire for sexual relations following treatment. There was no significant difference between those treated by XRT, XRT followed by salvage chemotherapy and those who received CMT ($P = 0.087$).

### *Finances and insurance*

A total of 362 patients answered a question regarding the long-term effects on finances and of these 53 (14.6%) reported that they were worse off financially as a result of their disease. A total of 174 patients had applied for life insurance following HD and 125 (71.8%) had experienced difficulty. Sixty-nine of those experiencing difficulty were eventually successful, but 29 believed that their premium had been excessively 'loaded'.

### *Employment*

A total of 192 men and 172 women responded to the questions concerning employment. Three men and one woman were unemployed at diagnosis of Hodgkin's disease and at the time of the questionnaire the corresponding figures were 12 men and 11 women. However, the total number of employed and homemakers before treatment was 293 and at the time of the study it was 300. The number of students fell from 60 to 5 and the number of retired rose from 7 to 32. Twenty-eight patients (7.8%) considered that their career had been greatly affected whereas 84 (23.5%) thought that it had been slightly affected by their illness.

### DISCUSSION

There have been many reports on late effects of treatment for Hodgkin's disease, mostly reporting on patients with all stages of disease. This study reports the results of an extensive review of late effects of treatment of a consecutive group of patients with stage I and II HD treated at a single institution. We address toxicities, including second malignancy and other illnesses, and also other effects such as exercise tolerance, psychological and sexual problems.

The increased risk of second malignancy after treatment of Hodgkin's disease has been recognized for more than two decades. The largest analysis of second malignancy was conducted by the International DataBase on Hodgkin's Disease (IDHD) (Henry-Amar, 1992) which includes the patients in the current study. There have been many others describing the relative risk of second malignancy as ranging from 1.86 to 6.8 and the 15-year incidences of second malignancy ranging from 11.2% to 18% (Hancock et al, 1988; Tucker et al, 1988; Cosset et al, 1991*a*; Henry-Amar, 1992; Swerdlow et al, 1992; Abrahamsen et al, 1993; Dietrich et al, 1994; van Leeuwen et al, 1994; Mauch et al, 1995; Bhatia et al, 1996). All studies showed a significant increased risk of second malignancies, in particular for acute leukaemia, non-Hodgkin's lymphoma and solid tumours. The relative risks of non-Hodgkin's lymphoma and leukaemia are considerably higher than for solid cancers because of a relative rarity of non-Hodgkin's lymphoma (NHL) and leukaemia compared with solid cancers. The Late Effects Study Groups (Bhatia et al, 1996) differs from the other studies as all patients entered were under the age of 16 at the time of treatment for Hodgkin's disease.

Several reports (Hancock et al, 1988; Rodriquez et al, 1993; Biti et al, 1994; Dietrich et al, 1994; van Leeuwen et al, 1994) indicated that age greater than 40 at the time of diagnosis was associated with an increased risk of second maligancies. Although the AR increased with age, because malignancy is more common with increasing age, the relative risk of developing a second malignancy was greater in younger patients in this study, as has been reported by others (Henry-Amar, 1992; Swerdlow et al, 1992). Splenectomy has been reported as a risk factor for second malignancy in some studies (van Leeuwen et al, 1994) but not others (Valagussa et al, 1986; Swerdlow et al, 1993). The IDHD reported a relative risk of 1.3 for development of acute leukaemia ($P < 0.05$) and 1.4 for non-Hodgkin's lymphoma ($P < 0.1$). Dietrich et al (1994), found that splenic irradiation as well as splenectomy was associated with an increased risk of development of second malignancy. We did not find a similar effect.

An increased risk of myocardial infarction has been reported following mediastinal radiation (Cosset et al, 1991*a*,*b*). Boivin et al (1992) reported an increased risk of mycoardial death at 5 years after radiation that persisted for 10 years or more, but was seen only in an early cohort of patients and not observed in the later cohort, treated by more modern radiotherapy techniques. Mauch et al (1995) reported a significantly increased cardiac mortality (RR 2.2, 1.2–3.6). In the current study there was an increased risk of death from myocardial infarction but it was not statistically significant (RR 1.5, CI 0.7–3.0). It is possible that the absence of an increased RR of death from myocardial infarction relates to the lower RT dose to the mediastinum and smaller RT fraction size (35 Gy in 1.75-Gy fractions) than is described by most of the other series. However, in the series reported from Stanford (Hancock et al, 1993), a persistent increased risk of myocardial infarction mortality was observed even after subcarinal blocking was introduced. The limited patient numbers and the relatively short length of follow-up in the current study may also contribute to the failure to find an increased risk of death from myocardial infarction.

Long-term pulmonary toxicity was difficult to assess by the questionnaire. It was difficult to exclude pre-existing pulmonary disease and difficult to differentiate between pneumonitis and pulmonary fibrosis. Accordingly, these were grouped together. Eight patients who were treated by radiation alone developed pneumonitis or pulmonary fibrosis and in three of these patients it was fatal; one patient had mediastinal reirradiation and another patient, who also developed radiation hepatitis, received standard mantle radiation to a dose of 35 Gy in 20 fractions and upper abdominal radiation with alternate fields per day. In comparison with the 3.75% incidence of pneumonitis in the current study, Tarbell et al (1990) reported a 6% incidence of pneumonitis in patients treated for stage 1A–3B Hodgkin's disease; the incidence was higher with patients treated with combined modality therapy, whole-lung irradiation and also in patients with large mediastinal adenopathy.

© Cancer Research Campaign 1998

Herpes zoster infection may develop within 2 years of treatment for Hodgkin's disease in 15–20% of patients (Hoppe, 1990). In a multi-institutional study of 717 patients with Hodgkin's disease (Guinee et al, 1985), the incidence was related to treatment intensity (11% for radiation alone and 27.5% for CMT) reflecting the findings of this study, in which the 15-year actuarial incidence was lower for radiation alone than for CMT. The study by Guinee et al(1983) found no influence on the attack rate of infections for stage, histology or splenectomy.

Many of the reports on thyroid function define hypothyroidism biochemically so that any patient with a raised thyroid-stimulating hormone (TSH) above the normal range is considered to have hypothyroidism. The rate of hypothyroidism in the current study is lower than that reported by other (Tarbell et al, 1990; Hancock et al, 1991; Peerboom et al, 1992) and could be due to the short follow-up or lower dose of radiation to the neck, or related to the differences in definitions of hypothyroidism. The Stanford series (Hancock et al, 1991) reported a higher risk of hypothyroidism in patients treated at a young age, which increased with length of follow-up and dose of radiation given. In the current study there were eight cases of Graves' disease, a high number, given the size of the study. Hancock et al (1991) reported an increased risk of Graves' disease of 7.2–20.4. Unlike Stanford, or the IDHD data, there were no thyroid cancers observed in our cohort.

That partners of male patients following treatment for Hodgkins have a lower frequency of pregnancy than female patients and that more men felt that they had been less successful in fathering pregnancies than they had wished and sought more medical advice with regards to fertility is in keeping with other reports (Aisner et al, 1993). Chapman et al (1981) and others (e.g. Viviani et al, 1991) reported that sperm counts may be reduced before treatment. It is therefore uncertain whether the reduction in fertility is related to the disease rather than the treatment. In the current study however, the reduction in numbers of wanted pregnancies tended to be higher in those treated with combined modality treatment than radiation alone, suggesting that the treatment was a relevant factor. Whether this effect will be observed in those receiving ABVD rather than MOPP remains to be seen.

Despite differences in allocation of modalities of treatment, stages of disease and types of questions posed, several reports including this study have demonstrated restriction in physical functioning, lower perceived overall health, less satisfaction with sexual life, marital difficulties, difficulty in obtaining financial loans and life insurance, and more health-related unemployment limitations in patients treated for Hodgkin's disease (Fobair et al, 1986; Kornblith et al, 1992*a, b*; Valagussa et al, 1992; van Tulder et al, 1994).

This study, in keeping with others, has documented an increase risk in second maliganancy following treatment for Hodgkin's disease. We did not show a significantly increased risk of cardiac disease. This may be related to the lower RT dose compared with most series, or to the small patient numbers and the short follow-up of this study. Change in chemotherapy practice, e.g. the increased use of ABVD, could affect the incidence of both cardiac and pulmonary disease with more prolonged follow-up. Changing combination chemotherapy regimens may, however, be associated with a reduction in the incidence of second malignancies, certainly reduced incidence of leukaemia and possibly reduced frequency of problems with fertility. In the meantime, care should continue to be given to optimize practice by the use of good radiotherapy techniques such as two fractions a day and keeping the total dose and fraction size to as small a dose as possible. This study has also demonstrated the psychological, social, financial and sexual problems that occur in patients following treatment for early Hodgkin's disease. It is difficult to know how much is related to treatment and how much is related to the disease itself. However, it seems unlikely that current changes in treatment management will have a profound impact on these deleterious effects. This study demonstrated the importance of careful follow-up after treatment for Hodgkin's disease. Consideration should be given to screening for the more common problems and counselling when necessary.

### REFERENCES

Abrahamsen JF, Andersen A, Hannisdal E, Nome O, Abrahamsen AF, Kvaloy S and Host H (1993) Second malignancies after treatment of Hodgkin's disease: the influence of treatment, follow-up time, and age. *J Clin Oncol* **11**: 255–261

Aisner J, Wiernik PH and Pearl P (1993) Pregnancy outcome in patients treated for Hodgkin's disease. *J Clin Oncol* **11**: 507–512

Bhatia S, Robison LL, Oberlin O, Greenberg M, Bunin G, Fossati-Bellani F and Meadows AT (1996) Breast cancer and other second neoplasms after childhood Hodgkin's disease. *N Engl J Med* **334**: 745–751

Biti G, Cellai E, Magrini SM, Papi MG, Ponticelli P and Boddi V (1994) Second solid tumors and leukemia after treatment for Hodgkin's disease: an analysis of 1121 patients from a single institution. *Int J Radiat Oncol Biol Phys* **29**: 25–31

Boivin J-F, Hutchison GB, Lubin JH and Mauch P (1992) Coronary artery disease mortality in patients treated for Hodgkin's disease. *Cancer* **69**: 1241–1247

Chapman RM, Sutcliffe SB, Rees LH, Edwards CRW and Malpas JS (1981) Male gonadal dysfunction in Hodgkin's disease: a prospective study. *JAMA* **245**: 1323–1328

Cosset JM, Henry-Amar M and Meerwaldt JH (1991*a*) Long-term toxicity of early stages of Hodgkin's disease therapy: the EORTC experience. *Ann Oncol* **2** (Suppl. 2): 77–82

Cosset JM, Henry-Amar M, Pellae-Cosset B, Carde P, Girinski T, Tubiana M and Hayat M (1991*b*) Pericarditis and myocardial infarctions after Hodgkin's disease therapy. *Int J Radiat Oncol Biol Phys* **21**: 447–449

Dietrich P-Y, Henry-Amar M, Cosset J-M, Bodis S, Bosq J and Hayat M (1994) Second primary cancers in patients continuously disease-free from Hodgkin's disease: a protective role for the spleen? *Blood* **84**: 1209–1215

Fobair P, Hoppe RT, Bloom J, Cox R, Varghese A and Spiegel D (1986) Psychosocial problems among survivors of Hodgkin's disease. *J Clin Oncol* **4**: 805–814

Gospodarowicz MK, Sutcliffe SB, Bergsagel DE and Chua T (1992*a*) Radiation therapy in clinical stage I and II Hodgkin's disease. *Eur J Cancer* **28A**: 1841–1846

Gospodarowicz MK, Sutcliffe SB, Clark RM, Dembo AJ, Fitzpatrick PJ, Munro AJ, Bergsagel DE, Patterson BJ, Tsang R, Chua T and Bush RS (1992*b*) Analysis of supradiaphragmatic clinical stage I and II Hodgkin's disease treated with radiation alone. *Int J Radiat Oncol Biol Phys* **22**: 859–865

Guinee VF, Guido JJ, Pfalzgraf KA, Giacco GG, Lagarde C, Durand M, van der Velden JW, Lowenberg B, Jereb B, Bretsky S, Meilof J, Hamersma EAM, Dische S and Anderson P (1985) The incidence of Herpes zoster in patients with Hodgkin's disease. *Cancer* **56**: 642–648

Hancock SL, Hoppe RT, Horning SJ and Rosenberg SA (1988) Intercurrent death after Hodgkin disease therapy in radiotherapy and adjuvant MOPP trials. *Ann Int Med* **109**: 183–189

Hancock SL, Cox RS and McDougall IR (1991) Thyroid diseases after treatment of Hodgkin's disease. *N Engl J Med* **325**: 599–605

Hancock SL, Tucker MA and Hoppe RT (1993) Factors affecting late mortality from heart disease after treatment of Hodgkin's disease. *JAMA* **270**: 1949–1955

Henry-Amar M (1992) Second cancer after the treatment for Hodgkin's disease: a report from the International Database on Hodgkin's disease. *Ann Oncol* **3** (Suppl. 4): S117–S128

Hoppe RT (1990) Radiation therapy in the management of Hodgkin's disease. *Sem Oncol* **17**: 704–715

Kaplan E and Meier P (1958) Nonparametric estimation from incomplete observations. *J Am Stat Assoc* **53**: 457–481

Kornblith AB, Anderson J, Cella DF, Tross S, Zuckerman E, Cherin E, Henderson E, Weiss RB, Cooper MR, Silver RT, Leone L, Canellos GP, Gottlieb A and

© Cancer Research Campaign 1998

Holland JC (1992*a*) Hodgkin's disease survivors at increased risk for problems in psychosocial adaptation. *Cancer* **70**: 2214–2224

Kornblith AB, Anderson J, Cella DF, Tross S, Zuckerman E, Cherin E, Henderson ES, Canellos GP, Kosty MP, Cooper MR, Weiss RB, Gottlieb A and Holland JC (1992*b*) Comparison of psychosocial adaptation and sexual function of survivors of advanced Hodgkin disease treated by MOPP, ABVD, or MOPP alternating with ABVD. *Cancer* **70**: 2508–2516

Mantel N (1966) Evaluation of survival data and two new rank order statistics arising in its consideration. *Cancer Chemother Rep* **50**: 163–170

Mauch PM, A KL, Marcus KC, Shulman LN, Krill E, Tarbell NJ, Silver B, Weinstein H, Come S, Canellos GP and Coleman N (1995) Long-term survival in Hodgkin's disease: relative impact of mortality, second tumors, infection, and cardiovascular disease. *Cancer J* **1**: 33–42

Peerboom PF, Hassink EAM, Melkert R, DeWit L, Nooijen WJ and Bruning PF (1992) Thyroid function 10–18 years after mantle field irradiation for Hodgkin's disease. *Eur J Cancer* **28A**: 1716–1718

Rodriquez MA, Fuller LM, Zimmerman SO, Allen PK, Brown BW, Munsell MF, Hagemeister FB, McLaughlin P, Velasquez WS, Swan Jr F and Cabanillas FF (1993) Hodgkin's disease: study of treatment intensities and incidences of second malignancies. *Ann Oncol* **4**: 125–131

Sutcliffe SB, Gospodarowicz MK, Bergsagel DE, Bush RS, Alison RE, Bean HA, Brown TC, Chua T, Clark RM, Curtis JE, Dembo AJ, Fitzpatrick PJ, Hasselback RH, Rideout DF, Sturgeon JF, Quirt I, Yeoh L and Peters MV (1985) Prognostic groups for management of localized Hodgkin's disease *J Clin Oncol* **3**: 393–401

Swerdlow AJ, Douglas AJ, Vaughan Hudson G, Vaughan Hudson B, Bennett MH and MacLennan KA (1992) Risk of second primary cancers after Hodgkin's disease by type of treatment: analysis of 2846 patients in the British National Lymphoma Investigation. *B M J* **304**: 1137–1143

Swerdlow AJ, Douglas AJ, Vaughan Hudson G, Vaughan Hudson B and MacLennan KA (1993) Risk of second primary cancer after Hodgkin's disease in patients in the British National Lymphoma Investigation: relationships to host factors, histology and stage of Hodgkin's disease, and splenectomy. *Br J Cancer* **68**: 1006–1011

Tarbell NJ, Thompson L and Mauch P (1990) Thoracic irradiation in Hodgkin's disease: disease control and long-term complications. *Int J Radiat Oncol Biol Phys* **18**: 275–281

Tucker MA, Coleman CN, Cox RS, Varghese A & Rosenberg SA (1988) Risk of second cancers after treatment for Hodgkin's disease. *N Engl J Med* **318**: 76–81

Valagussa P, Santoro A, Fossati-Bellani F, Banfi A and Bonadonna G (1986) Second acute leukemia and other malignancies following treatment for Hodgkin's disease. *J Clin Oncol* **4**: 830–837

Valagussa P, Santoro A and Bonadonna G (1992) Thyroid, pulmonary and cardiac sequelae after treatment for Hodgkin's disease. *Ann Oncol* **3**(Suppl. 4): S111–S115

van Leeuwen FE, Klokman WJ, Hagenbeek A, Noyon R, van den Belt-Dusebout AW, van Kerkhoff EHM, van Heerde P and Somers R (1994) Second cancer risk following Hodgkin's disease: a 20-year follow-up study. *J Clin Oncol* **12**: 312–325

van Tulder MW, Aaronson NK and Bruning PF (1994) The quality of life of long-term survivors of Hodgkin's disease. *Ann Oncol* **5**: 153–158

Viviani S, Ragni G, Santoro S, Perotti L, Caccamo E, Negretti E, Valagussa P and Bonadonna G (1991) Testicular dysfunction in Hodgkin's disease before and after treatment. *Eur J Cancer* **27**: 1389–1392

© Cancer Research Campaign 1998

### APPENDIX 1

### Questionnaire for female patients

Have you developed any of the following illnesses or conditions since you were treated for Hodgkin's disease?

| | | |
|---|---|---|
| Myocardial infarction (heart attack) | [ ] No [ ] Yes | |
| Other heart disease | [ ] No [ ] Yes | |
| Thyroid disease | [ ] No [ ] Yes | |
| Kidney disease | [ ] No [ ] Yes | |
| High blood pressure | [ ] No [ ] Yes | |
| Peptic ulcer | [ ] No [ ] Yes | [ ] Stomach<br>[ ] Duodenal<br>[ ] Don't know |
| Cancer, tumour, growth or leukaemia | [ ] No [ ] Yes | |
| Lung disease | [ ] No [ ] Yes | |
| Shingles*Herpes zoster* infection | [ ] No [ ] Yes | number of episodes_______________ |
| Liver disease | [ ] No [ ] Yes | |
| Blood disorder | [ ] No [ ] Yes | |
| Other medical problems | [ ] No [ ] Yes | Please specify_______________ |

Have you had any surgery since you were treated for Hodgkin's disease? [ ] No [ ] Yes Please specify_______________

Please choose the **best** description of your **current** physical condition.

[ ] normal, no complaints
[ ] able to carry on normal activities with only minor complaints
[ ] can manage normal activities but only with some effort
[ ] able to care for most of your own needs but require occasional assistance
[ ] require considerable assistance and frequent medical care
[ ] disabled and requiring special care and assistance

Do you think your HD or its treatment has resulted in any restriction in the amount of exercise you can take or your performance in sporting activities?

[ ] not at all restricted
[ ] a little restricted
[ ] quite a lot restricted
[ ] very restricted

**Before** you were treated for Hodgkin's disease, were you:

[ ] a student
[ ] employed
[ ] managing household
[ ] unemployed
[ ] retired
[ ] other. Please specify:

**At present** are you:

[ ] a student
[ ] employed
[ ] managing household
[ ] unemployed
[ ] retired
[ ] other. Please specify:

Does your current health **currently** keep you from working at a job? [ ] No [ ]Yes

Does your current health **currently** keep you from doing household jobs? [ ] No [ ] Yes

© Cancer Research Campaign 1998

How much has the Hodgkin's disease (and its treatment) interfered with progress in your career?
[ ] not interfered at all
[ ] interfered slightly
[ ] interfered greatly

Have you applied for life-insurance since you were found to have Hodgkin's disease?
[ ] No [ ] Yes did you experience any difficulty because of your Hodgkin's disease?
[ ] No [ ] Yes were you eventually successful?
[ ] No [ ] Yes has your premium been *loaded* because of your Hodgkin's disease? [ ] No [ ] Yes

Do you think that you are **currently** worse-off financially as a result of having had Hodgkin's disease? [ ] No [ ] Yes

When you were found to have Hodgkin's disease, were you:
(A) **Married** (or living as married)
[ ] No Please go to (**B**) below
[ ] Yes **1.** Have you:
[ ] Remained with the same partner
[ ] Divorced/separated and since remained single
[ ] Divorced/separated but now remarried (or living as married)
[ ] Other. Please specify:_______________

**2.** Do you think that having Hodgkin's disease has affected your marriage?
[ ] favourable
[ ] unfavourably
[ ] not at all

(**B**) **Unmarried** (or living as married)
[ ] Yes **1.** Have you:
[ ] Remained single
[ ] Married (or living as married)
[ ] Married (or living as married) temporarily, but now single again
[ ] Other. Please specify:_______________

**2.** Do you think that having Hodgkin's disease has affected your marriage prospects?
[ ] favorably
[ ] unfavorably
[ ] not at all

*What effect did the treatment for Hodgkin's disease have on your periods?
[ ] No periods at time of diagnosis and none since
[ ] Periods have continued
[ ] Periods stopped for a time, later restarted
How long did they stop for?____months
[ ] Periods stopped and never restarted
[ ] Periods continued for a while but later stopped
How old were you when they stopped?____years
[ ] Other. Please specify:

Have you been through the menopause yet?
[ ] No [ ] Yes Please state approximately how old you were when this took place____years

How many children did you have **at the time** your Hodgkin's disease was diagnosed?___________
Please complete results of any pregnancies you have had **since** your Hodgkin's disease was diagnosed:
a) Number of live babies
b) Number of stillborn babies
c) Number of miscarriages
d) Number of therapeutic abortions
e) Other. Please specify:_______________

© Cancer Research Campaign 1998

Since your treatment for Hodgkin's disease, have you sought medical advice regarding possible infertility?
[ ] No [ ] Yes Please specify________________________________

Have you had any children born with congenital abnormalities (birth defects)?
[ ] No [ ] Yes Please specify________________________________

Have any of your children developed any serious illnesses or conditions?
[ ] No [ ] Yes Please specify________________________________

Since your treatment for Hodgkin's disease, have you been successful in becoming pregnant as you wished?
[ ] No [ ] Yes Please specify________________________________

Did the Hodgkin's disease and its treatment cause any reduction in your interest in (desire for) sexual relations?
[ ] no reduction
[ ] temporary reduction
[ ] permanent reduction

How else has Hodgkin's disease and its treatment changed/interfered with your life? Please describe below:

*QUESTIONS ASKED OF MALE PATIENTS FROM THIS POINT

How many children did you have **at the time** you Hodgkin's disease was diagnosed?

How many pregnancies have you fathered **since** your Hodgkin's disease was diagnosed (including pregnancies which ended with miscarriages or abortions besides those which went to full term)?

Since your treatment for Hodgkin's disease, have you sought medical advice regarding possible infertility?
[ ] No [ ] Yes Please specify________________________________

Have you had any children born with congenital abnormalities (birth defects)?
[ ] No [ ] Yes Please specify________________________________

Have any of your children developed any serious illnesses or conditions?
[ ] No [ ] Yes Please specify________________________________

Since your treatment for Hodgkin's disease, have you been as successful in fathering pregnancies as you wished?
[ ] No [ ] Yes Please specify________________________________

Did the Hodgkin's disease and its treatment cause any reduction in your interest in (desire for) sexual relations?
[ ] No reduction
[ ] temporary reduction
[ ] permanent reduction

How else has Hodgkin's disease and its treatment changed/interfered with your life? Please describe below:

*© Cancer Research Campaign 1998*